# Multidrug Efflux Systems in Microaerobic and Anaerobic Bacteria

**DOI:** 10.3390/antibiotics4030379

**Published:** 2015-08-28

**Authors:** Zeling Xu, Aixin Yan

**Affiliations:** 1 School of Biological Sciences, The University of Hong Kong, Hong Kong Special Administrative Region, Hong Kong, China; E-Mail: zelingxu@hku.hk; 2 Institute of Scientific and Industrial Research, Osaka University, Yamadaoka 1-1, Suita, Osaka 565-0871, Japan

**Keywords:** multi-drug efflux pump, microaerobic bacteria, anaerobic bacteria, efflux pump inhibitors

## Abstract

Active drug efflux constitutes an important mechanism of antibiotic and multidrug resistance in bacteria. Understanding the distribution, expression, and physiological functions of multidrug efflux pumps, especially under physiologically and clinically relevant conditions of the pathogens, is the key to combat drug resistance. In animal hosts, most wounded, infected and inflamed tissues display low oxygen tensions. In this article, we summarize research development on multidrug efflux pumps in the medicinally relevant microaerobic and anaerobic pathogens and their implications in the effort to combat drug-resistant infections.

## 1. Introduction

Since the first antibiotic, penicillin, was put into clinical practice in the early 20th century, hundreds of antibiotics have been developed and applied in clinical medicine. However, bacterial infectious diseases remain a major problem in public health [1]. An important reason for this ineptness is the emergence and prevalence of bacterial multidrug resistance (MDR). Thus far, four general strategies bacteria utilize to develop resistance have been identified, prevention of drug entry, active drug efflux, modification of drug targets, and inactivation of drugs by hydrolyzation or enzymatic modification. Among them, active drug extrusion, which is carried out by a class of membrane proteins called multidrug efflux pumps, is the major reason for bacterial simultaneous resistance to multiple antibiotics, *i.e.*, multidrug resistance, and is frequently associated with clinically isolated resistance [2,3]. To combat multidrug resistance, it is necessary to investigate the distribution, expression, and physiological functions of multidrug efflux pumps in bacteria, especially under the physiologically and medically relevant conditions.

In animal hosts, most wounded, infected, and inflamed tissues display low oxygen tensions. Examples include inflamed gut, *Schistosoma mansoni*-infected tissue, *Streptococcus pyogenes*- and *Leishmania amazonensis*-infected skin, and *Mycobacterium tuberculosis*- or *Aspergillus fumigatus*-infected lung tissue [4]. In addition, facultative anaerobes including all major pathogens that infect the lower gastrointestinal (GI) tract constantly encounter oxygen fluctuations [5]. Although significant developments on the identification, expression, and physiological functions of bacterial multidrug efflux pumps under ordinary laboratory conditions and in model bacterial species have been achieved in the past decades, those in the physiologically and clinically relevant microaerobic and anaerobic niches of pathogens have not been extensively studied. In this article, we focus on the recent research development on bacterial multidrug efflux pumps in microaerobic and obligate anaerobic niches of pathogens (Table 1) and their implications in combating drug resistance.

## 2. Classification of Efflux Pumps and Their Regulation

Multidrug efflux pumps exist in almost all bacterial species. According to their different compositions, energy sources, substrates, and number of transmembrane domains, multidrug efflux pumps are divided into five classes: (i) the Major Facilitator Superfamily (MFS); (ii) the Resistance-Nodulation-Division (RND) family; (iii) the ATP-Binding Cassette (ABC) superfamily; (iv) the Small Multidrug Resistance (SMR) family; and (v) the Multidrug And Toxic compound Extrusion (MATE) family [1,6,7]. In addition to antibiotics, multidrug efflux pumps are also found to expel a broad range of environmental and physiological toxic compounds, such as dyes, detergents, bile acids, hormones, organic acids, *etc.* Thus, they are also referred to as xenobiotic pumps or transporters.

**Figure 1:**
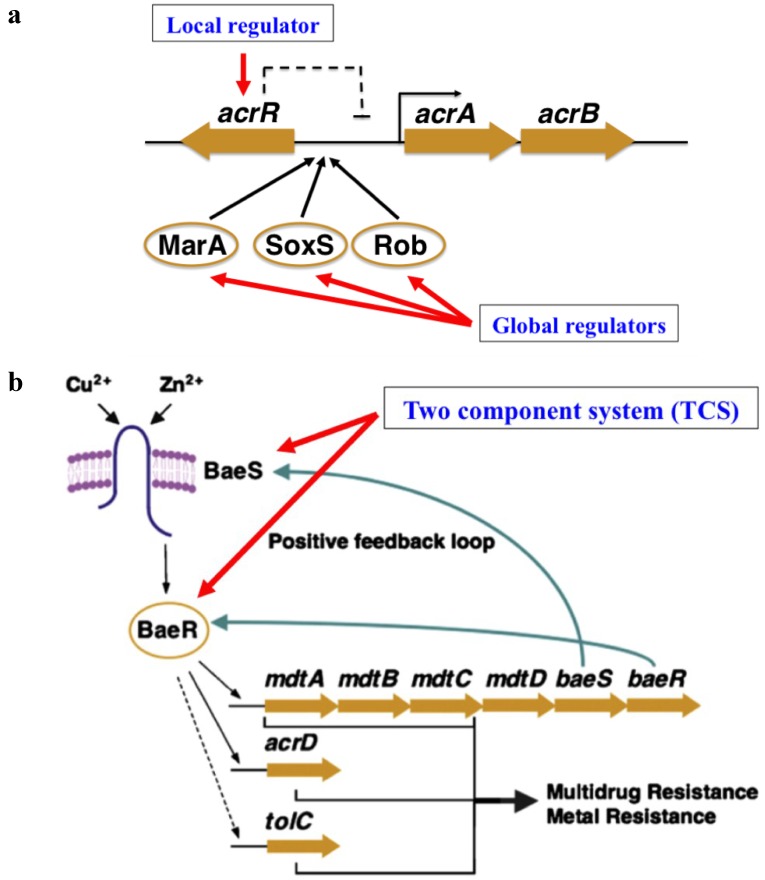
Regulatory patterns of multidrug efflux pumps (using those in *Salmonella typhimurium* as an example). (**a**) Local repressor and global regulators: local regulator AcrR inhibits the expression of *acrAB* operon and global regulators MarA, SoxS, and Rob act as inducers of *acrAB* in response to different signals; (**b**) Two component system (TCS): BaeRS activates expression of the *mdt* operon, *acrD*, and *tolC*. Figures are adopted and modified based on those by Nishino *et al.* and Blair *et al.* [33,34].

Although ubiquitously encoded in bacterial genomes, except for a handful of housekeeping systems, the production levels of the majority of efflux pumps are low and their expression is often under the strict control of multiple regulators. Currently, efflux pump regulators are mainly categorized into three groups (Figure 1) [6]: (i) local repressors, which usually are located adjacent to the regulated efflux genes to repress their expression. They often belong to the MarR, MerR, or TetR transcription factor superfamily, such as AcrR, which represses the expression of *acrAB* in *Salmonella* [8,9,10]; (ii) global response regulators, which are often activated in response to certain stimuli and regulate a large scale of gene expression in addition to that of efflux pumps such as SoxS, which regulates *acrAB* expression in response to superoxide stress in *Salmonella* [11]; and (iii) two component systems (TCSs), which are consisted of a sensor kinase and a cognate response regulator. Examples include the BaeSR TCS, which activates the expression of *mdtABCD* [12,13]. All these three regulatory patterns which have been identified in the efflux pump expression in aerobic bacteria are also present in microaerobic and anaerobic pathogens.

**Table 1:** A summary of the distribution, substrate spectrum, and regulation of bacterial efflux systems in microaerobic or anaerobic niches.

Species	Major Efflux Pumps	Regulators	Antimicrobial Agents Being Pumped	Reference
Microaerobic niches				
*Campylobacter* spp*.*	CmeABC (RND)	CmeR (TetR)	Ciprofloxacin, Norfloxacin, Cefotaxime, Fusidic Acid, Erythromycin	[14,15,16]
		CosR (OmpR)		
	CmeDEF (RND)		Ampicillin, Polymyxin B, Ethidium Bromide	[14]
	CmeG (MFS)		Ciprofloxacin, Erythromycin, Gentamicin, Tetracycline, Rifampicin, Ethidium Bromide, Cholic Acid, Hydrogen Peroxide	[17]
	NhaA1/NhaA2 (cation/proton antiporters)		Trisodium Phosphate	[18]
*Helicobacter pylori*	HefABC (RND)		Metronidazole, Tetracycline, Erythromycin, Penicillin G, Ciprofloxacin	[19,20]
	HefDEF (RND)			[19]
	HefGHI (RND)			[19]
*Staphylococcus aureus*	NorB	MgrA (MarR)	Moxifloxacin, Sparfloxacin	[21]
Anaerobic niches				
*Bacteroides fragilis*	BmeABC1-16 (RND)	BmeR (TetR)	Cephems, Polypeptide Antibiotics, Fusidic Acid, Novobiocin, Puromycin Ampicillin, Cefoxitin, Cefoperazone, Ciprofloxacin, Metronidazole, Imipenem, Ethidium Bromide, Sodium Dodecyl Sulfate.	[22,23]
	BexA		Fluoroquinolone	[24]
*Clostridium perfringens*	bcrABD	bcrR	Phenotypic Bacitracin	[25]
*Escherichia coli*	MdtEF (RND)	ArcAB	Indole Nitrosative Derivatives erythromycin	[26,27]
		(TCS)		
		MnmE		
		H-NS		
	CusCBA (RND)	CusSR (TCS)	Cu(I)	[28]
*Porphyromonas gingivalis*	XepCAB (RND)		Rifampin, Puromycin, Ethidium Bromide	[29]
*Salmonella enterica*	AcrAB (RND)			[30]
	TolC			[31]
	TetA		Etracycline, Ethidium Bromide	[31]
*Staphylococcus aureus*	MnhF		Bile Salts	[32]

## 3. Drug Efflux Pumps in Microaerobic Niches

### 3.1. Campylobacter spp. Efflux Pumps

*Campylobacter* spp. are the major foodborne, gram-negative, microaerophilic pathogens that can cause diarrhea, abdominal cramps, and fever in humans [35]. Antibiotics such as erythromycin or ciprofloxacin are utilized to treat severe cases, although the infections of *Campylobacter* are often clinically mild and self-limiting [36]. In the past decades, however, *Campylobacter* spp. Isolates, which display high tolerance to multiple antibiotics, have been isolated and the resistance was found to be largely achieved by multidrug efflux pumps [37].

CmeABC and CmeDEF are the two major RND efflux systems responsible for intrinsic resistance in *Campylobacter* *jejuni* (*C*. *jejuni*) (Table 1), the most common human pathogen among *Campylobacter* species. Both of them have been reported to contribute to the resistance of *C. jejuni* to various antibiotics such as fluoroquinolones, macrolide, and tetracycline [14,38,39]. Expression of *cmeABC* was reported to be under the control of CmeR, a repressor belonging to the TetR family transcription factors. CmeR binds to the promoter region of *cmeABC*, specifically to the inverted repeat (IR) sequences, and represses its transcription. *cmeR* deletion mutant was shown to display high resistance to various antibiotics such as ciprofloxacin, norfloxacin, cefotaxime, erythromycin, and fusidic acid (two- to four-fold in MICs) [15]. Another regulator named CosR, an OmpR-type oxidative stress response regulator, was also found to be a repressor of *cmeABC*, and *cosR* deletion also led to high transcriptional levels of *cmeABC* [16,40]*.* It has been found that the binding of CmeR to the promoter region of *cmeABC* can be inhibited by bile salts [41]. Since bile salts are the detergent molecules generated by the liver and secreted into the small intestine in the host that are harmful to bacteria, the increased *cmeABC* expression by bile salts not only promoted the survival of *C*. *jejuni* in the human intestinal tract but also led to enhanced resistance to multiple antibiotics [41,42]. Salicylate, which is often present in humans as the major metabolite of aspirin, a widely used medicine, was also reported to be able to induce the expression of *cmeABC* by inhibiting the function of CmeR in *C. jejuni* NCTC 11168 and cause resistance to fluoroquinolones [43,44].

In addition to CmeABC and CmeDEF, other efflux systems existing in *C*. *jejuni* were also found to contribute to antibiotic resistance (Table 1) [37]. For example, CmeG, a putative efflux pump of the MFS family, was found to be involved in antibiotic resistance and oxidative stress response in *C*. *jejuni*. A *cmeG* deletion mutant showed increased susceptibility to a broad range of antibiotics such as ciprofloxacin, erythromycin, gentamicin, tetracycline, *etc.* [17]. In addition, NhaA1/NhaA2 cation/proton antiporters and several as-yet uncharacterized RND multidrug efflux pumps were found to contribute to the tolerance of *C*. *jejuni* to sub-lethal concentrations of trisodium phosphate (TSP) [18], an antimicrobial agent that can disrupt the cytoplasmic and outer membranes of cells and is used frequently for food decontamination [45,46].

### 3.2. Helicobacter pylori Efflux Pumps

*Helicobacter* *pylori* (*H*. *pylori*) is another common gram-negative, microaerophilic pathogen that inhabits the human stomach and duodenum [37]. *H*. *pylori* is associated with various diseases including chronic gastritis, peptic ulcers, low-grade gastric mucosa-associated lymphoid tissue (MALT) lymphoma, noncardiac gastric adenocarcinoma, and other gut diseases [47]. Different kinds of antibiotics have been used to treat *H*. *pylori* infection*.* Among those, clarithromycin (CLR) was considered as the most common and effective drug. Unfortunately, antibiotic resistance is increasingly being identified in *H*. *pylori* and efflux pumps were reported to play an important role [48,49].

Studies revealed that *H*. *pylori* contains three putative RND efflux operons, namely *hefABC*, *hefDEF*, and *hefGHI* (Table 1) [19]*.* However, so far most studies have focused on *hefA* and *hefC*. An amoxicillin-resistant *Helicobacter* *hepaticus* (*H*. *hepaticus*) strain demonstrated a bile salt-induced resistance profile similar to that which occurs in *C*. *jejuni* [41,50]. Such induction resulted in antibiotic resistance in *H*. *hepaticus* and its adaptation to the host intestinal tract [50]. In addition to bile salts, expression of *hefA* could also be induced by other antibiotics, resulting in efflux-mediated resistance to different antibiotics. For example, the relative expression level of *hefA* in chloramphenicol-induced *H*. *pylori* strains was found to be higher than that in the wild-type strain, and MICs of these strains to metronidazole, tetracycline, erythromycin, penicillin G, and ciprofloxacin were significantly increased (≥four-fold) [20]. In another *H*. *pylori* strain that was exposed to metronidazole (Mtz), *hefA* expression was also induced significantly. This may explain the high Mtz resistance among clinical isolates of *H*. *pylori* due to the frequent application of Mtz in medicine [51]. *H*. *pylori* efflux pumps were also proposed to be involved in resistance to CLR, since the typical efflux pumps inhibitor (EPI) Phe-Arg β-naphthylamide dihydrochloride (PaβN) reduced the MIC of 14 out of 15 *H*. *pylori* strains analyzed to CLR with a four-fold or greater extent in the studies carried out by Kenro *et* *al.* [49]. Furthermore, it was found that biofilm could induce the expression of efflux pump genes and consequently enhance the resistance of *H*. *pylori* to CLR, since compared to the planktonic cells, a four-fold elevation of MIC to CLR was observed in *H*. *pylori* TK1402 biofilms [52]. Since biofilm of *H*. *pylori* has been detected in the gastric mucosa of certain patients infected with the pathogen [53], this result implied that specific efflux inhibition might need to be considered in the treatment of the infection.

### 3.3. Staphylococcus aureus Efflux Pumps

*Staphylococcus* *aureus* (*S*. *aureus*) is a gram-positive, facultative bacterium which can grow under oxygen-limited condition by fermentation or nitrate respiration [54]. It can cause a broad range of infectious diseases in humans, ranging from minor skin infections such as pimples, to life-threatening diseases such as pneumonia, toxic shock syndrome (TSS), blood infections, and sepsis. The emergence of antibiotic-resistant *S*. *aureus* pathogen, e.g., Methicillin-Resistant *Staphylococcus* *aureus* (MRSA), which is associated with increased expression of *norA*, has been a worldwide problem in clinical medicine. It was reported that in *S*. *aureus* N315, there are at least 30 genes encoding putative drug efflux pumps, and most of them are major facilitator superfamily (MFS) proteins (Table 1) [55].

Given that in hosts different infection sites have different oxygen tensions (for example, the abscess of skin infection can create completely anaerobic conditions), studies to examine gene expression changes in *S*. *aureus* under aerobic and anaerobic conditions have been conducted [54]. MgrA, a MarR family transcription factor, is a global regulator of *S*. *aureus* that negatively controls the expression of efflux pump genes *norB* and *norC* [56]. It was demonstrated that under reduced aeration, the dimeric DNA-binding form of MgrA was disrupted by posttranslational modification and only the monomeric form was observed. Consequently, *norB* transcription was de-repressed and the resistance of *S*. *aureus* to moxifloxacin and sparfloxacin (four-fold in MICs) was elevated [21]. Furthermore, deletion of *mgrA* was also found to promote the formation of biofilm [57], which might further enhance efflux-mediated multidrug resistance under anaerobic condition [52]. Under anaerobic conditions, it was reported that MnhF, a mammalian bile salt transporter homologue, was induced and contributed to the resistance of *S*. *aureus* to bile salts in the host [32]. *mnhF* deletion mutant led to decreased survival of *S*. *aureus* in an anaerobic three-stage continuous-culture model of the human colon (gut model) [32,58].

## 4. Drug Efflux Pumps in Anaerobic Niches

*Bacteroides* *fragilis* (*B*. *fragilis*) is a common obligate anaerobe, which is usually considered as a friendly commensal in human. However, it is also the most commonly isolated anaerobic pathogen [59,60]. In recent years, increasing cases of MDR *B*. *fragilis* have been reported worldwide [61].

Among the different factors that cause multidrug resistance in *B*. *fragilis*, efflux pumps were proposed to be one of the culprits, because in the case of fluoroquinolones, treatment with efflux pump inhibitor carbonyl cyanide m-chlorophenylhydrazone (CCCP) led to a significant (five-fold) increase of ciprofloxacin accumulation in *B*. *fragilis* [62]. Subsequently, 16 homologues of *P*. *aeruginosa* MexAB-OprM system were identified in *B*. *fragilis* and named BmeABC1–16 (Table 1), where A, B, and C represent the membrane fusion protein, cytoplasmic efflux transporter protein, and outer membrane channel protein, respectively [22]. It was found that all 16 genes of *bmeB1–16* were constitutively expressed in *B*. *fragilis* and at least seven efflux pumps were responsible for transporting antibiotics with overlapping substrates [63]. Using the BmeABC5 system as an example, BmeR5 was proposed as a TetR family regulator that could specifically bind to the *bmeR5–bmeC5* intergenic region (IT1) to repress the expression of *bmeABC5* operon [23]. A *bmeR5* deletion mutation led to increased expression of *bmeA5*, *bmeB5*, and *bmeC5*, and two-fold increase in MICs to multiple chemicals such as ampicillin, cefoxitin, cefoperazone, ciprofloxacin, imipenem, metronidazole, ethidium bromide (EB), and sodium dodecyl sulfate (SDS) [23]. In addition to Bme, 7.5% of moxifloxacin-resistant *B*. *fragilis* strains isolated from different European countries were found to possess additional efflux pump BexA to extrude fluoroquinolone [24].

## 5. Physiological Roles of Efflux Pumps during the Anaerobic Adaptation of Facultative Bacteria

Facultative bacterial pathogens can live under both low and high oxygen tensions and consequently are present in a broad range of human infections. Efflux systems in several of the model organisms in this group, such as *Escherichia* *coli*, *Salmonella* *enterica* serovar Typhimurium, *Pseudomonas* *aeruginosa*, *etc.*, have been studied extensively. In this section, we focus on those efflux systems whose expression is altered in response to low oxygen tension in the anaerobic life style of these bacteria.

### 5.1. Escherichia coli Efflux Pumps

The model organism *Escherichia* *coli* (*E*. *coli*), a gram-negative facultative anaerobe, contains at least 20 drug efflux systems [64]. Except for the housekeeping efflux pump AcrAB-TolC, expression of the rest of the efflux systems is low under ordinary laboratory conditions with sufficient oxygen. Studies from our group demonstrated that under anaerobic conditions, the typical environment of the human gut, expression of the RND efflux pump MdtEF was induced more than 20-fold and the induction was dependent on the anaerobic global regulator ArcA and a GTP binding protein MnmE [26,27]. It was shown that ArcA antagonized H-NS-mediated repression of the *gadE-mdtEF* operon and consequently increased its expression [27]. Interestingly, this anaerobically upregulated expression of MdtEF promoted the growth of *E*. *coli* during anaerobic respiration of nitrate by removing nitrosyl indole derivatives, a metabolic byproduct generated under this physiological condition, and simultaneously enhanced the resistance of *E*. *coli* to erythromycin and Rhodamine 6G [26].

In addition to antibiotics, metal ions represent another class of ancient antimicrobial agent and metal efflux systems are broadly distributed in bacterial genomes or plasmids. Recently, transition metals were also reported to act as an important immune defense strategy used by the animal host to combat pathogens [65]. For example, it was demonstrated that copper concentration was increased at pulmonary tissues infected by *Mycobacterium* *tuberculosis* (*M*. *tuberculosis*) [66]. Copper could also delay the growth of *Rhodopseudomonas* *palustris* TIE-1 under anaerobic conditions synergistically with iron [67]. In addition, copper supplementation could protect dairy cattle from *E*. *coli*-induced mastitis [68]. In *E*. *coli*, copper can cause cellular toxicity by eliciting a Fenton reaction, which results in the production of Reactive Oxygen Species (ROS) and by damaging the iron–sulfur [Fe-S] cluster enzymes [65,69]. The RND pump CusCBA is dedicated to copper efflux in *E*. *coli*. Studies from our group have demonstrated that CusCBA was induced under anaerobic amino acid limited conditions without exogenous Cu supplement. The induced CusCBA protected Fe-S cluster enzymes and their biosynthesis to facilitate bacterial adaptation to this physiological condition and led to enhanced Cu and Ag tolerance in the bacterium under this condition [28].

### 5.2. Salmonella enterica Efflux Pumps

*Salmonella* *enterica* serovar Typhimurium (*S*. *enterica* serovar Typhimurium) is a pathogen of human and animals that possesses nine potential drug transporters associated with drug resistance [70]. As in *E*. *coli*, AcrAB is the housekeeping RND family efflux pump that confers resistance to a variety of antibiotics, dyes, and detergents (Table 1) [71,72]. The AcrAB-TolC efflux pump has also been reported to be involved in invasion of host epithelial cells and macrophages *in* *vitro*, and colonization of the bacterium in poultry [73]. Under aerobic conditions, it has been reported that several regulators including AcrR, MarA, SoxS, and RamA can regulate the expression of the AcrAB-TolC efflux pump [74,75,76]. Expression of the efflux systems under anaerobic conditions has not been studied. However, it was reported that under anaerobic conditions, deletion of *acrA*, *acrB*, and *tolC* affected transcription of 115, 569, and 171 genes, respectively, including those involved in pathogenicity, thereby indicating the relationship between anaerobic adaptation, antibiotics resistance, and pathogenicity [30]. It was also reported that expression of the multidrug efflux protein TolC and tetracycline efflux protein TetA were induced when *S*. *enterica* serovar Typhimurium CCARM 8009 was exposed to bile salts under anaerobic conditions, which resulted in enhanced antibiotic resistance and pathogenesis [31].

### 5.3. Role of Efflux Pumps in the Anaerobic Niches of Bacterial Communities

Another ecological and physiological environment with significant low oxygen tension is bacterial biofilm, which is defined as a matrix-enclosed bacterial population adherent to an abiotic or biotic surface [77]. Oxygen concentration decreases from the top of the biofilm to the bottom and this oxygen limitation in one species’ biofilm can promote the growth of other anaerobic bacteria when they coexist in the oxic areas of the host [78]. In the community of bacterial biofilms, a diverse range of metabolites are produced and secreted, including certain “antibiotics,” which belong to a class of microbial secondary metabolites produced by certain species but cytotoxic to the surrounding species. These “antibiotics” often have important physiological roles for those species that produce them [79]. For example, phenazines, a class of secondary metabolite produced primarily by the species of *Pseudomonads*, were recently found to play important roles in signaling, iron acquisition, community development, and anaerobic survival of *P*. *aeruginosa* [79,80,81]. Two such phenazine compounds, phenazine-1-carboxylic acid (PCA) and pyocyanin (PYO), have been shown to facilitate the biofilm formation and anaerobic growth of *P. aeruginosa* [82]. Although it has not been proved directly, efflux pumps are speculated to play a role in exporting these antibiotic phenazines out of producing cells, allowing their redox exchange with extracellular compounds to promote electron shuttling and iron acquisition [81]. A promising efflux pump for this physiological role is the MexGHI-OpmD in *P. aeruginosa* PAO1 since genes encoding this system are located immediately downstream of the phenazine biosynthetic operon in the bacterium and their expression is induced by PYO [83].

## 6. Other Efflux Systems

Expression of efflux pumps and their contribution to antibiotic resistance in the anaerobic niches of several other species have also been explored (Table 1). For instance, in the gram-negative pathogen *Pseudomonas aeruginosa* (*P. aeruginosa*), it was reported that anaerobic incubation increased MICs of the bacterium to tobramycin, amikacin, and aztreonam by approximately seven-, four-, and six-fold, respectively, compared to under aerobic condition [84], which was proposed to be mediated by efflux pump systems. Studies that analyzed the expression of the membrane fusion protein component of the RND efflux system under anaerobic condition proposed that anaerobiosis-induced antibiotic resistance might be due to the alteration of the expression of membrane fusion protein, which shifted the formation of RND efflux pumps to a dominance of the active MexEF-OprN pump [85]. In the dairy species *Streptococcus* *thermophiles*, the efflux pump TetA was reported to play a role in detoxification of several cytotoxic compounds, including tetracycline and ethidium bromide [86]. In *Porphyromonas* *gingivalis*, a gram-negative obligate anaerobe, a RND family efflux pump XepCAB was responsible for transporting multiple agents including rifampin, puromycin, and ethidium bromide and enhanced the resistance of the bacterium to these compounds [29]. In *Clostridium* *perfringens*, the *bcrABD* operon, together with its regulator *bcrR*, was shown to contribute to the resistance to bacitracin, which was confirmed by using EPI thioridazine [25]. Another obligate anaerobic pathogenic species, *Prevotella* spp., also contains RND family efflux pumps that enhance their resistance to tetracycline [87].

## 7. Inhibition of Drug Efflux in Microaerobic and Anaerobic Niches

Considerable studies have demonstrated that bacterial multidrug efflux pumps play important roles in the drug resistance of pathogens in their microaerobic and anaerobic niches. Given that most infection sites in hosts are hypoxic [4], it is necessary to develop efflux pump inhibitors (EPIs) to restrain efflux activities under this physiological environment [88]. It was reported that *C*. *jejuni* NCTC11168 treated by PaβN decreased its resistance to trisodium phosphate (TSP) [18]. Given that CmeABC in *C*. *jejuni* mediates resistance to multiple drugs, inhibition of CmeABC using efflux pump inhibitor (EPI) PaβN serves as a potential means to decrease the efflux pump-associated antibiotic resistance [89]. EPIs have also been shown to be effective against multidrug-resistant *H*. *pylori*. It was demonstrated that antibiotic susceptibilities of a chloramphenicol-induced, *hefA*-dependent multidrug resistant *H*. *pylori* strain could be partially restored by carbonyl cyanide m-chlorophenyl hydrazone (CCCP) and pantoprazole, two different types of EPIs. A four- to 19-fold decrease in the MICs of a *H*. *pylori* strain to nine tested antibiotics such as erythromycin, ciprofloxacin, tetracycline, *etc.* was observed following the treatment of these two EPIs [90]. Recently, four chemicals from herbal extracts, namely emodin, baicalin, schizandrin, and berberine, were reported to reduce the resistance of *H*. *pylori* to amoxicillin and tetracycline (one- to two-fold) through inhibition of *hefA* expression [91], indicating a novel intervention in drug-resistant infections using natural products.

## 8. Conclusions

Antibiotic resistance has been a public health threat worldwide. Drug efflux constitutes an important mechanism of multidrug resistance that is frequently isolated from clinical settings. In the past several decades, significant advancement in the distribution, expression, and substrate profiles of drug efflux pumps has been achieved [6]. However, less attention has been focused on the study of the pathogens present in microaerobic and strict anaerobic niches, which represent the conditions in wounded, infected, and inflamed tissues within a human host [4].

The fact that efflux genes ubiquitously exist in bacterial genomes prior to antibiotic development and application suggested that they have physiological functions and efflux-mediated drug resistance is perhaps merely a side effect of their over-expression under certain inducing conditions. Indeed, increasing evidence on the roles of efflux pumps in the general detoxification and physiological adaptation in bacteria has been reported in recent years. Specific physiological roles of efflux pumps in microaerobic and anaerobic pathogens are also starting to be revealed (Table 1). Understanding their regulation and physiological functions not only promotes our understanding of the emergence and prevalence of drug resistance but also their prediction and prevention. Furthermore, the role of efflux in bacterial communities represents an emerging research topic in the area, given that naturally produced antibiotics not only can inhibit bacterial growth but also can function as signals to affect the establishment and maintenance of community dynamics in complex environments, especially at low concentrations [92]. An interesting example is indole, a common secondary metabolite produced by *E. coli* recently found to be able to serve as a signal to turn on the efflux pumps of antibiotic-susceptible sister cells and promote the survival of the entire population [93]. Study of efflux pump expression in the context of populations such as in biofilms or polymicrobial communities, as well as in the presence of antibiotics, is necessary to explore their corresponding physiological significances.

In recent decades, efflux pump inhibitors (EPIs) have emerged as a potential approach to circumvent efflux activities by means of energy supply disruption, antagonizing the substrate binding, substrates modification, *etc.* [88]. A number of EPIs have been intensively studied but no clinical EPIs are available currently [94]. In addition to EPIs, antibiotic drugs ineffectual against specific antibiotic-resistant pathogens could be considered as a second choice behind clearing other types of pathogens in order to preserve the current antibiotics pipeline. For example, fosfomycin and rifampin have been recently reported to have a better effect on eradicating *H*. *pylori* than CLR and Mtz, owing to their lower MICs [95]. Further studies on both the mechanisms of antibiotic resistance and the development of EPIs using synthetic tools and natural sources, especially under physiologically and clinically relevant conditions, are required to combat antibiotic and multidrug resistance.

## Abbreviations

ABC: the ATP (adenosine triphosphate)-binding cassette superfamily
CCCP: carbonyl cyanide m-chlorophenylhydrazone
CLR: clarithromycin
EB: ethidium bromide
EPI: efflux pump inhibitor
MATE: the multidrug and toxic compound extrusion family
MFS: the major facilitator superfamily
MIC: minimum inhibitory concentration
Mtz: metronidazole
PaβN: Phe-Arg β-naphthylamide dihydrochloride
RND: the resistance-nodulation-division family
SDS: sodium dodecyl sulfate
SMR: the small multidrug resistance family
TCS: two component system
TSP: trisodium phosphate

## References

[1] Poole K. (2007). Efflux pumps as antimicrobial resistance mechanisms. Ann. Med..

[2] Hirakawa H., Inazumi Y., Senda Y., Kobayashi A., Hirata T., Nishino K., Yamaguchi A. (2006). *N*-Acetyl-d-Glucosamine induces the expression of multidrug exporter genes, *mdtEF*, via catabolite activation in *Escherichia*
*coli*. J. Bacteriol..

[3] Piddock L.J. (2004). Multidrug-resistance efflux pumps—Not just for resistance. Nat. Rev. Microbiol..

[4] Jantsch J., Schodel J. (2015). Hypoxia and hypoxia-inducible factors in myeloid cell-driven host defense and tissue homeostasis. Immunobiology.

[5] Marteyn B., Scorza F.B., Sansonetti P.J., Tang C. (2011). Breathing life into pathogens: The influence of oxygen on bacterial virulence and host responses in the gastrointestinal tract. Cell Microbiol..

[6] Sun J., Deng Z., Yan A. (2014). Bacterial multidrug efflux pumps: Mechanisms, physiology and pharmacological exploitations. Biochem. Biophys. Res. Commun..

[7] Putman M., Veen H.W., Konings W. (2000). Molecular properties of bacterial multidrug transporters. Microbiol. Mol. Biol. Rev..

[8] Perera I.C., Grove A. (2010). Molecular mechanisms of ligand-mediated attenuation of DNA binding by MarR family transcriptional regulators. J. Mol. Cell Biol..

[9] Brown N.L., Stoyanov J.V., Kidd S.P., Hobman J.L. (2003). The MerR family of transcriptional regulators. FEMS Microbiol. Rev..

[10] Ramos J.L., Martinez-Bueno M., Molina-Henares A.J., Teran W., Watanabe K., Zhang X., Gallegos M.T., Brennan R., Tobes R. (2005). The TetR family of transcriptional repressors. Microbiol. Mol. Biol. Rev..

[11] Nikaido E., Shirosaka I., Yamaguchi A., Nishino K. (2011). Regulation of the AcrAB multidrug efflux pump in *Salmonella*
*enterica* serovar Typhimurium in response to indole and paraquat. Microbiology.

[12] Hoch J.A. (2000). Two-component and phosphorelay signal transduction. Curr. Opin. Microbiol..

[13] Baranova N., Nikaido H. (2002). The BaeSR two-component regulatory system activates transcription of the *yegMNOB* (*mdtABCD*) transporter gene cluster in *Escherichia*
*coli* and increases its resistance to novobiocin and deoxycholate. J. Bacteriol..

[14] Akiba M., Lin J., Barton Y.W., Zhang Q. (2006). Interaction of CmeABC and CmeDEF in conferring antimicrobial resistance and maintaining cell viability in *Campylobacter*
*jejuni*. J. Antimicrob. Chemoth..

[15] Lin J., Akiba M., Sahin O., Zhang Q. (2005). CmeR functions as a transcriptional repressor for the multidrug efflux pump CmeABC in *Campylobacter*
*jejuni*. Antimicrob. Agents Chemother..

[16] Hwang S., Zhang Q., Ryu S., Jeon B. (2012). Transcriptional regulation of the CmeABC multidrug efflux pump and the KatA catalase by CosR in *Campylobacter*
*jejuni*. J. Bacteriol..

[17] Jeon B., Wang Y., Hao H., Barton Y.W., Zhang Q. (2011). Contribution of CmeG to antibiotic and oxidative stress resistance in *Campylobacter*
*jejuni*. J. Antimicrob. Chemother..

[18] Riedel C.T., Cohn M.T., Stabler R.A., Wren B., Brondsted L. (2011). Cellular Response of *Campylobacter*
*jejuni* to Trisodium Phosphate. Appl. Environ. Microbiol..

[19] Bina J.E., Alm R.A., Uria-nichelsen M., Thomas S.R., Trust T.J., Hancock R.E.W. (2000). *Helicobacter pylori* uptake and efflux: Basis for intrinsic susceptibility to antibiotics *in*
*vitro*. Antimicrob. Agents Chemother..

[20] Liu Z.Q. (2008). Efflux pump gene hefA of *Helicobacter*
*pylori* plays an important role in multidrug resistance. World J. Gastroenterol..

[21] Truong-Bolduc Q.C., Hsing L.C., Villet R., Bolduc G.R., Estabrooks Z., Taguezem G.F., Hooper D.C. (2012). Reduced aeration affects the expression of the NorB efflux pump of *Staphylococcus*
*aureus* by posttranslational modification of MgrA. J. Bacteriol..

[22] Ueda O., Wexler H.M., Hirai K., Shibata Y., Yoshimura F., Fujimura S. (2005). Sixteen homologs of the mex-type multidrug resistance efflux pump in *Bacteroides*
*fragilis*. Antimicrob. Agents Chemother..

[23] Pumbwe L., Chang A., Smith R.L., Wexler H.M. (2007). BmeRABC5 is a multidrug efflux system that can confer metronidazole resistance in *Bacteroides*
*fragilis*. Microbiol. Drug Resist..

[24] Eitel Z., Soki J., Urban E., Nagy E. (2013). Infection ESGoA. The prevalence of antibiotic resistance genes in *Bacteroides*
*fragilis* group strains isolated in different European countries. Anaerobe.

[25] Charlebois A., Jalbert L.A., Harel J., Masson L., Archambault M. (2012). Characterization of genes encoding for acquired bacitracin resistance in *Clostridium*
*perfringens*. PLoS ONE.

[26] Zhang Y., Xiao M., Horiyama T., Zhang Y., Li X., Nishino K., Yan A. (2011). The multidrug efflux pump MdtEF protects against nitrosative damage during the anaerobic respiration in *Escherichia*
*coli*. J. Biol. Chem..

[27] Deng Z., Shan Y., Pan Q., Gao X., Yan A. (2013). Anaerobic expression of the gadE-mdtEF multidrug efflux operon is primarily regulated by the two-component system ArcBA through antagonizing the H-NS mediated repression. Front. Microbiol..

[28] Fung D.K.C., Lau W.Y., Chan W.T., Yan A. (2013). Copper efflux is induced during anaerobic amino acid limitation in *Escherichia*
*coli* to protect iron-sulfur cluster enzymes and biogenesis. J. Bacteriol..

[29] Ikeda T., Yoshimura F. (2002). A resistance-nodulation-cell division family xenobiotic efflux pump in an obligate anaerobe, *Porphyromonas*
*gingivalis*. Antimicrob. Agents Chemother..

[30] Webber M.A., Bailey A.M., Blair J.M., Morgan E., Stevens M.P., Hinton J.C., Ivens A., Wain J., Piddock L.J. (2009). The global consequence of disruption of the AcrAB-TolC efflux pump in *Salmonella*
*enterica* includes reduced expression of SPI-1 and other attributes required to infect the host. J. Bacteriol..

[31] He X., Ahn J. (2013). Assessment of efflux-mediated antibiotic-resistant *Salmonella*
*enterica* serovar Typhimurium under simulated gastrointestinal conditions. Ann. Microbiol..

[32] Sannasiddappa T.H., Hood G.A., Hanson K.J., Costabile A., Gibson G.R., Clarke S.R. (2015). *Staphylococcus*
*aureus* MnhF mediates cholate efflux and facilitates survival under human colonic conditions. Infect Immun..

[33] Nishino K., Nikaido E., Yamaguchi A. (2009). Regulation and physiological function of multidrug efflux pumps in *Escherichia*
*coli* and *Salmonella*. Biochim. Biophys. Acta.

[34] Blair J.M., Webber M.A., Baylay A.J., Ogbolu D.O., Piddock L.J. (2015). Molecular mechanisms of antibiotic resistance. Nat. Rev. Microbiol..

[35] Blaser M.J. (1997). Epidemiologic and clinical features of *Campylobacter*
*jejuni* infections. J. Infect. Dis..

[36] Bolton D., Patriarchi A., Fox Á., Fanning S. (2013). A study of the molecular basis of quinolone and macrolide resistance in a selection of *Campylobacter* isolates from intensive poultry flocks. Food Control..

[37] Li X., Nikaido H. (2009). Efflux-mediated drug resistance in bacteria: An update. Drugs.

[38] Jeon B., Zhang Q. (2009). Sensitization of *Campylobacter*
*jejuni* to fluoroquinolone and macrolide antibiotics by antisense inhibition of the CmeABC multidrug efflux transporter. J. Antimicrob. Chemoth..

[39] Gibreel A., Wetsch N.M., Taylor D.E. (2007). Contribution of the CmeABC efflux pump to macrolide and tetracycline resistance in *Campylobacter*
*jejuni*. Antimicrob. Agents Chemother..

[40] Hwang S., Kim M., Ryu S., Jeon B. (2011). Regulation of oxidative stress response by CosR, an essential response regulator in *Campylobacter*
*jejuni*. PLoS ONE.

[41] Lin J., Cagliero C., Guo B., Barton Y.W., Maurel M.C., Payot S., Zhang Q. (2005). Bile salts modulate expression of the CmeABC multidrug efflux pump in *Campylobacter*
*jejuni*. J. Bacteriol..

[42] Thanassi D.G., Cheng L.W., Nikaido H. (1997). Active efflux of bile salts by *Escherichia*
*coli*. J. Bacteriol..

[43] Hartog E., Menashe O., Kler E., Yaron S. (2010). Salicylate reduces the antimicrobial activity of ciprofloxacin against extracellular *Salmonella*
*enterica* serovar Typhimurium, but not against *Salmonella* in macrophages. J. Antimicrob. Chemother..

[44] Shen Z., Pu X.Y., Zhang Q. (2011). Salicylate functions as an efflux pump inducer and promotes the emergence of fluoroquinolone-resistant *Campylobacter*
*jejuni* mutants. Appl. Environ. Microbiol..

[45] Oyarzabal O.A. (2005). Reduction of *Campylobacter* spp. by commercial antimicrobials applied during the processing of broiler chickens: A review from the United States Perspective. J. Food Protect..

[46] Sampathkumar B., Khachatourians G.G., Korber D.R. (2003). High pH during trisodium phosphate treatment causes membrane damage and destruction of *Salmonella*
*enterica* Serovar Enteritidis. Appl. Environ. Microbiol..

[47] Suzuki H., Hibi T., Marshall B.J. (2007). *Helicobacter*
*pylori*: Present status and future prospects in Japan. J. Gastroenterol..

[48] Qureshi W.A., Graham D.Y. (2000). Antibiotic-resistant *H*. *pylori* infection and its treatment. Curr. Pharm. Design..

[49] Hirata K., Suzuki H., Nishizawa T., Tsugawa H., Muraoka H., Saito Y., Matsuzaki J., Hibi T. (2010). Contribution of efflux pumps to clarithromycin resistance in *Helicobacter pylori*. J. Gastroen. Hepatol..

[50] Belzer C., Stoof J., Breijer S., Kusters J.G., Kuipers E.J., van Vliet A.H.M. (2009). The *Helicobacter*
*hepaticus* hefA gene is involved in resistance to amoxicillin. Helicobacter.

[51] Tsugawa H., Suzuki H., Muraoka H., Ikeda F., Hirata K., Matsuzaki J., Saito Y., Hibi T. (2011). Enhanced bacterial efflux system is the first step to the development of metronidazole resistance in *Helicobacter pylori*. Biochem. Biophys. Res. Commun..

[52] Yonezawa H., Osaki T., Hanawa T., Kurata S., Ochiai K., Kamiya S. (2013). Impact of *Helicobacter*
*pylori* biofilm formation on clarithromycin susceptibility and generation of resistance mutations. PLoS ONE.

[53] Carron M.A., Tran V.R., Sugawa C., Coticchia J.M. (2006). Identification of *Helicobacter*
*pylori* biofilms in human gastric mucosa. J. Gastrointest. Surg..

[54] Fuchs S., Pane-Farre J., Kohler C., Hecker M., Engelmann S. (2007). Anaerobic gene expression in *Staphylococcus aureus*. J. Bacteriol..

[55] Kosmidis C., Schindler B.D., Jacinto P.L., Patel D., Bains K., Seo S.M., Kaatz G.W. (2012). Expression of multidrug resistance efflux pump genes in clinical and environmental isolates of *Staphylococcus*
*aureus*. Int. J. Antimicrob. Agents.

[56] Truong-Bolduc Q.C., Strahilevitz J., Hooper D.C. (2006). NorC, a new efflux pump regulated by MgrA of *Staphylococcus*
*aureus*. Antimicrob. Agents Chemother..

[57] Trotonda M.P., Tamber S., Memmi G., Cheung A.L. (2008). MgrA Represses biofilm formation in *Staphylococcus*
*aureus*. Infect Immun..

[58] Sannasiddappa T.H., Costabile A., Gibson G.R., Clarke S.R. (2011). The influence of *Staphylococcus*
*aureus* on gut microbial ecology in an *in*
*vitro* continuous culture human colonic model system. PLoS ONE.

[59] Teixeira F.L., Silva D.N., Pauer H., Ferreira L.Q., Ferreira Ede O., Domingues R.M., Lobo L.A. (2013). The role of BmoR, a MarR family regulator, in the survival of *Bacteroides*
*fragilis* during oxidative stress. Int. J. Med. Microbiol..

[60] Wexler H.M. (2007). Bacteroides: The good, the bad, and the nitty-gritty. Clin. Microbiol. Rev..

[61] Urban E., Horvath Z., Soki J., Lazar G. (2015). First Hungarian case of an infection caused by multidrug-resistant *Bacteroides*
*fragilis* strain. Anaerobe.

[62] Herin O., Hedberg M., Edlund C. (2002). Efflux-mediated fluoroquinolone resistance in the *Bacteroides*
*fragilis* Group. Anaerobe.

[63] Pumbwe L., Ueda O., Yoshimura F., Chang A., Smith R.L., Wexler H.M. (2006). *Bacteroides*
*fragilis* BmeABC efflux systems additively confer intrinsic antimicrobial resistance. J. Antimicrob. Chemother..

[64] Nishino K., Yamaguchi A. (2001). Analysis of a complete library of putative drug transporter genes in *Escherichia*
*coli*. J. Bacteriol..

[65] Braymer J.J., Giedroc D.P. (2014). Recent developments in copper and zinc homeostasis in bacterial pathogens. Curr. Opin. Chem. Biol..

[66] Hood M.I., Skaar E.P. (2012). Nutritional immunity: Transition metals at the pathogen-host interface. Nat. Rev. Microbiol..

[67] Bird L.J., Coleman M.L., Newman D.K. (2013). Iron and copper act synergistically to delay anaerobic growth of bacteria. Appl. Environ. Microbiol..

[68] White C., Lee J., Kambe T., Fritsche K., Petris M.J. (2009). A role for the ATP7A copper-transporting ATPase in macrophage bactericidal activity. J. Biol. Chem..

[69] Macomber L., Imlay J.A. (2009). The iron-sulfur clusters of dehydratases are primary intracellular targets of copper toxicity. Proc. Natl. Acad. Sci. USA.

[70] Nishino K., Latifi T., Groisman E.A. (2006). Virulence and drug resistance roles of multidrug efflux systems of *Salmonella*
*enterica* serovar Typhimurium. Mol. Microbiol..

[71] Eaves D.J., Ricci V., Piddock L.J.V. (2004). Expression of acrB, acrF, acrD, marA, and soxS in *Salmonella*
*enterica* Serovar Typhimurium: Role in multiple antibiotic resistance. Antimicrob. Agents Chemother..

[72] Ricci V., Tzakas P., Buckley A., Piddock L.J. (2006). Ciprofloxacin-resistant *Salmonella*
*enterica* serovar Typhimurium strains are difficult to select in the absence of AcrB and TolC. Antimicrob. Agents Chemother..

[73] Buckley A.M., Webber M.A., Cooles S., Randall L.P., la Ragione R.M., Woodward M.J., Piddock L.J. (2006). The AcrAB-TolC efflux system of *Salmonella*
*enterica* serovar Typhimurium plays a role in pathogenesis. Cell Microbiol..

[74] Nikaido E., Yamaguchi A., Nishino K. (2008). AcrAB multidrug efflux pump regulation in *Salmonella*
*enterica* serovar Typhimurium by RamA in response to environmental signals. J. Biol. Chem..

[75] Balleste-Delpierre C., Sole M., Domenech O., Borrell J., Vila J., Fabrega A. (2014). Molecular study of quinolone resistance mechanisms and clonal relationship of *Salmonella*
*enterica* clinical isolates. Int. J. Antimicrob. Agents.

[76] Olliver A., Valle M., Chaslus-Dancla E., Cloeckaert A. (2004). Role of an acrR mutation in multidrug resistance of *in*
*vitro*-selected fluoroquinolone-resistant mutants of *Salmonella*
*enterica* serovar Typhimurium. FEMS Microbiol. Lett..

[77] Costerton J.W., Lewandowski Z., Caldwell D.E., Korber D.R., Lappin-Scott H.M. (1995). Microbial biofilms. Annu. Rev. Microbiol..

[78] Fox E.P., Cowley E.S., Nobile C.J., Hartooni N., Newman D.K., Johnson A.D. (2014). Anaerobic bacteria grow within *Candida*
*albicans* biofilms and induce biofilm formation in suspension cultures. Curr. Biol..

[79] Wang Y., Kern S.E., Newman D.K. (2010). Endogenous phenazine antibiotics promote anaerobic survival of *Pseudomonas*
*aeruginosa* via extracellular electron transfer. J. Bacteriol..

[80] Price-Whelan A., Dietrich L.E.P., Newman D.K. (2006). Rethinking “secondary” metabolism: Physiological roles for phenazine antibiotics. Nat. Chem. Biol..

[81] Dietrich L.E.P., Teal T.K., Price-Whelan A., Newman D.K. (2008). Redox-active antibiotics control gene expression and community behavior in divergent bacteria. Science.

[82] Wang Y., Wilks J.C., Danhorn T., Ramos I., Croal L., Newman D.K. (2011). Phenazine-1-carboxylic acid promotes bacterial biofilm development via ferrous iron acquisition. J. Bacteriol..

[83] Dietrich L.E.P., Price-Whelan A., Petersen A., Whiteley M., Newman D.K. (2006). The phenazine pyocyanin is a terminal signalling factor in the quorum sensing network of *Pseudomonas aeruginosa*. Mol. Microbiol..

[84] King P., Citron D.M., Griffith D.C., Lomovskaya O., Dudley M.N. (2010). Effect of oxygen limitation on the *in*
*vitro* activity of levofloxacin and other antibiotics administered by the aerosol route against *Pseudomonas*
*aeruginosa* from cystic fibrosis patients. Diagn. Micr. Infec. Dis..

[85] Schaible B., Taylor C.T., Schaffer K. (2012). Hypoxia increases antibiotic resistance in *Pseudomonas*
*aeruginosa* through altering the composition of multidrug efflux pumps. Antimicrob. Agents Chemother..

[86] Arioli S., Guglielmetti S., Amalfitano S., Viti C., Marchi E., Decorosi F., Giovannetti L., Mora D. (2014). Characterization of tetA-like gene encoding for a major facilitator superfamily efflux pump in *Streptococcus*
*thermophilus*. FEMS Microbiol. Lett..

[87] Sherrard L.J., Schaible B., Graham K.A., McGrath S.J., McIlreavey L., Hatch J., Wolfgang M.C., Muhlebach M.S., Gilpin D.F., Schneiders T. (2014). Mechanisms of reduced susceptibility and genotypic prediction of antibiotic resistance in *Prevotella* isolated from cystic fibrosis (CF) and non-CF patients. J. Antimicrob. Chemother..

[88] Bhardwaj A.K., Mohanty P. (2012). Bacterial efflux pumps involved in multidrug resistance and their inhibitors: rejuvinating the antimicrobial chemotherapy. Recent Pat. Antiinfect. Drug Discov..

[89] Lin J., Martinez A. (2006). Effect of efflux pump inhibitors on bile resistance and *in*
*vivo* colonization of *Campylobacter*
*jejuni*. J. Antimicrob. Chemother..

[90] Zhang Z. (2010). Influence of efflux pump inhibitors on the multidrug resistance of *Helicobacter*
*pylori*. World J. Gastroenterol..

[91] Huang Y.Q., Huang G.R., Wu M.H., Tang H.Y., Huang Z.S., Zhou X.H., Yu W.Q., Su J.W., Mo X.Q., Chen B.P. (2015). Inhibitory effects of emodin, baicalin, schizandrin and berberine on hefA gene: Treatment of *Helicobacter*
*pylori*-induced multidrug resistance. World J. Gastroenterol..

[92] Yim G., McClure J., Surette M.G., Davies J.E. (2011). Modulation of *Salmonella* gene expression by subinhibitory concentrations of quinolones. J. Antibiot..

[93] Lee H.H., Molla M.N., Cantor C.R., Collins J.J. (2010). Bacterial charity work leads to poplation-wide resistance. Nature.

[94] Nakashima R., Sakurai K., Yamasaki S., Hayashi K., Nagata C., Hoshino K., Onodera Y., Nishino K., Yamaguchi A. (2013). Structural basis for the inhibition of bacterial multidrug exporters. Nature.

[95] Boyanova L., Davidkov L., Gergova G., Kandilarov N., Evstatiev I., Panteleeva E., Mitov I. (2014). *Helicobacter*
*pylori* susceptibility to fosfomycin, rifampin, and 5 usual antibiotics for *H*. *pylori* eradication. Diagn. Micr. Infec. Dis..

